# Epidermal growth factor receptor mutations and anaplastic lymphoma kinase rearrangements in lung cancer with nodular ground-glass opacity

**DOI:** 10.1186/1471-2407-14-312

**Published:** 2014-05-03

**Authors:** Sung-Jun Ko, Yeon Joo Lee, Jong Sun Park, Young-Jae Cho, Ho Il Yoon, Jin-Haeng Chung, Tae Jung Kim, Kyung Won Lee, Kwhanmien Kim, Sanghoon Jheon, Hyojin Kim, Jae Ho Lee, Choon-Taek Lee

**Affiliations:** 1 Division of Pulmonary and Critical Care Medicine, Department of Internal Medicine, Seoul National University Bundang Hospital, Seongnam, Korea; 2 Department of Internal Medicine, Seoul National University Bundang Hospital, 173-82 Gumi-Ro, Bundang-Gu, Seongnam 464-707, Korea; 3 Department Pathology, Seoul National University College of Medicine, Seongnam, Korea; 4 Department of Radiology, Seoul National University Bundang Hospital, Seongnam, Korea; 5 Department of Thoracic and Cardiovascular Surgery, Seoul National University Bundang Hospital, Seongnam, Korea; 6 Department of Pathology, Seoul National University Hospital, Seoul, Korea

**Keywords:** Lung cancer, Adenocarcinoma, nGGO, *ALK*, *EGFR*

## Abstract

**Background:**

Nodular ground-glass opacities (nGGO) are a specific type of lung adenocarcinoma. *ALK* rearrangements and driver mutations such as *EGFR* and *K-ras* are frequently found in all types of lung adenocarcinoma. *EGFR* mutations play a role in the early carcinogenesis of nGGOs, but the role of *ALK* rearrangement remains unknown.

**Methods:**

We studied 217 nGGOs resected from 215 lung cancer patients. Pathology, tumor size, tumor disappearance rate, and the *EGFR* and *ALK* markers were analyzed.

**Results:**

All but one of the resected nGGOs were adenocarcinomas. *ALK* rearrangements and *EGFR* mutations were found in 6 (2.8%) and 119 (54.8%) cases. The frequency of *ALK* rearrangement in nGGO was significantly lower than previously reported in adenocarcinoma. Advanced disease stage (p = 0.018) and larger tumor size (p = 0.037) were more frequent in the *ALK* rearrangement-positive group than in *ALK* rearrangement-negative patients. nGGOs with *ALK* rearrangements were associated with significantly higher pathologic stage and larger maximal and solid diameter in comparison to *EGFR*-mutated lesions.

**Conclusion:**

*ALK* rearrangement is rare in lung cancer with nGGOs, but is associated with advanced stage and larger tumor size, suggesting its association with aggressive progression of lung adenocarcinoma. *ALK* rearrangement may not be important in early pathogenesis of nGGO.

## Background

Low-dose chest computed tomography (CT) for lung cancer screening has increased the detection of solitary pulmonary nodules (SPN) not visualized on chest radiography, and has contributed to a reduction in lung cancer mortality [1]. Some of these visualized nodules are nodular ground-glass opacities (nGGOs). nGGOs on chest CT are defined as hazy, increased attenuation of the lung with preservation of bronchial and vascular margins, and are classified as pure and mixed GGOs, which contain a solid component [2].

Nodular GGOs can be found in eosinophilic lung disease, pulmonary lymphoproliferative disorder, and interstitial fibrosis, with a persistent nGGO being a possible sign of early lung cancer [3]. The natural development of nGGO follows a stepwise progression from atypical adenomatous hyperplasia (AAH) to adenocarcinoma *in situ* (AIS: formerly bronchioloadenocarcinoma), to microinvasive adenocarcinoma (MIA), and finally to invasive adenocarcinoma (IA) [4]. However, some adenocarcinomas do not follow this pathway, manifesting as consolidation and/or solid mass, with different genetic profiles. Therefore, lung adenocarcinoma exhibits heterogeneity in pathogenesis and progression [5].

Several driver mutations have been identified in lung cancer, such as *epidermal growth factor receptor (EGFR)* and *K-ras* mutations and *anaplastic lymphoma kinase* (*ALK*) rearrangement. Lung cancers expressing *EGFR* mutations respond well to the EGFR tyrosine kinase inhibitors [6-8]. The fusion of echinoderm microtubule-associated protein-like 4 (*EML4*) and *ALK* gene by rearrangement in non-small cell lung cancer was identified [9] and developed as a target of the ALK tyrosine kinase inhibitor, crizotinib [10,11]. These biomarkers predict response to these molecular targeting agents and testing for these markers is recommended in lung cancer patients [12,13], enabling personalized medicine for patients harboring *EGFR* mutations or *ALK* gene rearrangements. It is therefore very important to investigate the frequencies and clinical implications of these driver mutations in nGGOs, a specific type of lung adenocarcinoma.

Many studies have reported that *EGFR* mutations are frequent in lung cancer with nGGOs, even in precancerous lesions such as AAH [14-17]; however, the role of *ALK* rearrangement in nGGOs remains unknown. We analyzed patients with lung cancer with nodular GGOs to investigate the correlation between biomarker status and clinicopathological and radiologic characteristics and to determine the roles of *ALK* rearrangements and *EGFR* mutations in nGGOs.

## Methods

### Patients

Among the patients who underwent surgical resection of their CT-identified nGGOs between August 2008 and March 2013 at Seoul National University Bundang Hospital (SNUBH), we selected patients who were diagnosed with lung cancer by pathologic confirmation of the surgical specimen. Multiple nGGOs in a single patient were considered different cases of nGGO. Patient data were extracted from medical records, including those pertaining to the age at the time of surgery, sex, smoking history quantified by packs per year, tumor histology, pathologic tumor stage, and biomarker status. This study was approved and individual patient consent waived by the institutional review board of Seoul National University Bundang Hospital (B-1305-202-102).

### Radiologic evaluation

Chest CT scans were performed preoperatively in each patient. All CT images were reviewed with a pulmonary window setting (window width, 2000 HU; window level, -500 HU) and mediastinal window setting (window width 440 HU, window level 45 HU). GGOs appear in pulmonary window images of chest CT, but disappear on mediastinal window images [3]. We included all nodules that contained any amount of GGO.

To evaluate the proportion of the solid component in the nGGOs, we measured the maximum transverse diameter (Tmax) and maximum perpendicular diameter (Pmax) of both the pulmonary and mediastinal window settings (pTmax, mTmax, pPmax, mPmax) and calculated the tumor shadow disappearance rate (TDR) in all nGGOs. TDR was calculated using the following formula: TDR = 1 - (mTmax × mPmax /pTmax × pPmax) [18].

### Histopathology review

Surgical specimens were reviewed by an experienced pathologist (J-H Chung) and another pathologist (H Kim). TNM classification was performed according to the Union for International Cancer Control and the American Joint Committee on Cancer staging system, 7th edition [19]. In some participants, lymph node dissection was not performed because lymphatic invasion was deemed unlikely in the preoperative evaluation; these participants were considered N0 stage. Lung cancer was histologically classified as adenocarcinoma or squamous cell carcinoma. The majority of participants were diagnosed with adenocarcinoma and were categorized according to the 2011 International Association for the Study of Lung Cancer/American Thoracic Society/European Respiratory Society (IASLC/ATS/ERS) classification system as adenocarcinoma *in situ* (AIS), minimally invasive adenocarcinoma (MIA), and various types of invasive adenocarcinoma (IA) [4].

### Molecular analysis

We analyzed the samples for *EGFR* mutation and *ALK* rearrangements. Genomic DNA was extracted from formalin-fixed paraffin-embedded specimens. Exons 18–21 of the *EGFR* gene were analyzed by PCR amplification and sequencing with an ABI Prism 3100 DNA analyzer and standard protocols. Peptide nucleic acid (PNA)-mediated PCR clamping or pyrosequencing methods are more sensitive than direct sequencing (DS) for *EGFR* mutation detection [20], but we have found that all of these methods are appropriate when sufficient tumor cells are properly micro-dissected and analyzed within a meticulously controlled turnaround time at a single institute (SNUBH) [21]. We included only nGGO specimens resected en bloc to ensure sufficient tumor cell sampling; this is the main strength of this study, as it provided highly accurate DS detection of *EGFR* mutations.

To detect *ALK* rearrangements, we first screened the tissues by immunohistochemistry (IHC) with monoclonal anti-*ALK* antibody (clone 5A4, Novocastra, 1:30, Newcastle, UK) and classified them with a four-tiered scoring system: 0, +1, +2, and +3. For cases with IHC scores of +2 or +3, fluorescence *in situ* hybridization (FISH) was used to detect *ALK* translocation by previously reported methods [22,23]. Concordance between IHC and FISH is high; thus, it is appropriate to use the sensitive IHC method for screening and FISH as a standard diagnostic test to detect *ALK* rearrangements [24].

### Statistical analysis

Statistical analysis was performed in SPSS version 18.0 for Windows (SPSS Inc., Chicago, IL). Numerical variables are expressed as mean ± standard deviation. All statistical tests were two-sided, and differences were considered statistically significant at P < 0.05.

## Results

### Patient characteristics

We recruited 289 patients who underwent surgical treatment for nGGOs from August 2009 to March 2013 at SNUBH. After pathologic confirmation of the surgical specimens, nine patients were excluded with diagnoses of non-cancerous lung conditions, including three interstitial fibroses, two lymphoplasma cell infiltrations, two chronic inflammations, one anthracofibrotic nodule, and one AAH. The remaining 280 nGGOs in 261 patients were considered lung cancer, including adenocarcinoma, squamous cell carcinoma, and adenosquamous carcinoma. We excluded 63 nGGOs in 46 patients for whom *EGFR* and/or *ALK* status was unavailable. Finally, 217 nGGO lesions in 215 patients were enrolled. Two patients had multiple nGGO lesions, which were tested for biomarker status. All nodules were diagnosed as adenocarcinoma, except one, which was identified as adenosquamous carcinoma.

### Pathologic classification of GGO nodules

Pathologic findings of 217 nGGOs were classified according to the 2011 IASLC/ATS/ERS classification. Numbers of AIS, MIA, and IA were 15, 16, and 185, respectively, and there was one adenosquamous carcinoma. Acinar predominant adenocarcinoma was the most frequent type in nGGOs. Seven solid predominant adenocarcinomas and five invasive mucinous adenocarcinomas also presented as nodules with GGOs. Six *ALK* rearrangement-positive (ALK-positive) nGGOs were invasive adenocarcinomas, whereas 11.8% (14 out of 119) of *EGFR* mutation-positive nGGOs were pre-invasive or minimally invasive adenocarcinomas. Subtypes of invasive adenocarcinoma revealed no statistical difference between *ALK* rearrangement and *EGFR* mutation-positive nGGOs (Table 1).

**Table 1:** Pathologic classification of GGO nodules according to the IASLC/ATS/ERS criteria, 2011

	**Number**	***ALK *** **positive**	***EGFR *** **positive**
Total	217	6	119
Adenocarcinoma *in situ*	15	0	3
Minimally invasive adenocarcinoma	16	0	11
Invasive adenocarcinoma			
Lepidic predominant	36	1	19
Acinar predominant	93	3	53
Papillary predominant	42	1	28
Micropapillary predominant	1	0	1
Solid predominant	7	1	3
Variants of invasive adenocarcinoma			
Invasive mucinous adenocarcinoma	5	0	0
Enteric	1	0	0
Adenosquamous carcinoma	1	0	1

### Analysis of ALK- and EGFR mutation-positive nodules

FISH identified *ALK* rearrangements in six lesions (2.8%) and *EGFR* mutations in 119 lesions (54.8%). These driver gene mutations were mutually exclusive in the examined nGGOs.

#### ALK-positive GGO nodules

Histopathology revealed that patients with *ALK*-positive nGGOs exhibited more advanced disease stages according to the AJCC, 7th edition (p = 0.018) (Table 2). *ALK*-positive nodules were significantly larger than *ALK*-negative nodules (p = 0.037). The solid proportion of *ALK*-positive nodules was also significantly larger than that of *ALK*-negative nodules (p = 0.039). All *ALK*-positive nodules were IA according to the 2011 IASLC/ATS/ERS classification; three nGGOs were acinar predominant subtypes, one was the solid subtype, one was the lepidic subtype, and one was the papillary predominant subtype (Table 1). Three nodules showed cribriform features and one nodule showed a signet ring cell pattern.

**Table 2:** **Clinicopathological characteristics according to *ALK *** **rearrangement status**

	***ALK *** **positive**	***ALK *** **negative**	**P value**
N	6	211	
Age	60.00 ± 12.05	63.22 ± 10.13	0.579
Sex (M:F)	2:4	96:115	0.692
PYR	0.750 ± 1.173	9.769 ± 17.381	0.464
Pathologic stage	6	206*	0.018
0	0	15	
IA	1	143	
IB	3	33	
IIA	1	5	
IIB	0	2	
IIIA	1	7	
IIIB	0	1	
Nodal involvement	2	13	0.060
Histologic invasiveness	6	210†	0.554
AIS	0	15	
MIA	0	16	
IA	6	179	
Maximal diameter	33.583 ± 13.736	22.528 ± 10.690	0.037
Solid diameter	23.217 ± 16.906	11.452 ± 10.920	0.039
TDR	0.533 ± 0.327	0.700 ± 0.290	0.209

*Data for pathologic stage were unavailable for 5 patients.

†Data for histologic invasiveness were unavailable for 1 patient.

#### EGFR mutation-positive GGO nodules

*EGFR* mutations were more frequent in women (p = 0.004) and in non-smokers or light smokers (p < 0.001). nGGOs with *EGFR* mutations did not significantly non-mutated lesions in terms of nodule size, solid proportion, nodal involvement, pathologic stage, and histologic invasiveness (Table 3). Among nGGO lesions with *EGFR* mutations, 56 nodules had a point mutation in exon 21 (L858R mutation in 54, L861Q in 1, and G863C in 1). Patients with *EGFR* mutations in exon 21 were older than patients with wild-type *EGFR* lesions (p = 0.034), were more likely to be non-smokers or light smokers (p = 0.002), and were more frequently women (p = 0.001). Patients with *EGFR* mutations in exons 19 or 20 showed no significant clinicopathological and radiologic differences in comparison to those without *EGFR* mutations (Table 4).

**Table 3:** **Clinicopathological characteristics according to *EGFR *** **mutation status**

	***EGFR *** **positive**	***EGFR *** **negative**	**P value**
N	119	98	
Age	63.50 ± 9.11	62.68 ± 11.35	0.559
Sex (M:F)	43:76	55:43	0.003
PYR	5.805 ± 14.426	14.031 ± 19.193	<0.001
Pathologic stage	117*	95*	0.199
0	6	9	
IA	87	57	
IB	18	18	
IIA	1	5	
IIB	2	0	
IIIA	3	5	
IIIB	0	1	
Nodal involvement	5	10	0.106
Histologic invasiveness	118†	98	0.600
AIS	6	9	
MIA	11	5	
IA	101	84	
Maximal diameter	22.387 ± 9.876	22.376 ± 12.052	0.507
Solid diameter	11.133 ± 11.229	12.559 ± 11.257	0.353
TDR	0.702 ± 0.295	0.687 ± 0.290	0.720

*Data for pathologic stage were unavailable for 2 EGFR positive and 3 EGFR negative patients.

†Data for histologic invasiveness were unavailable for 1 patient.

**Table 4:** **Clinicopathological characteristics according to *EGFR *** **mutation type**

	***EGFR *** **exon 19**	***EGFR *** **exon 20**	***EGFR *** **exon 21**	**EGFR negative**
N	50	9	56 (L858R in 54)	98
Age	60.40 ± 8.83	64.22 ± 8.04	66.45 ± 8.80**	62.68 ± 11.35
Sex	M:F = 23:27	M:F = 4:5	M:F = 16:40**	55:43
PYR	8.09 ± 14.90	2.72 ± 6.55	4.68 ± 15.25**	14.03 ± 19.19
Pathologic stage	50	9	54*	95
0	4	0	2	9
IA	40	6	38	57
IB	4	2	12	18
IIA	0	0	1	5
IIB	1	1	0	0
IIIA	1	0	1	5
IIIB	0	0	0	1
Nodal involvement	1	1	2	10
Histologic invasiveness	50	9	55†	98
AIS	4	0	2	9
MIA	7	0	4	5
IA	39	9	49	84
Maximal diameter	21.294 ± 10.713	26.944 ± 12.692	22.950 ± 8.769	22.376 ± 12.052
Solid diameter	9.392 ± 11.754	16.489 ± 15.322	11.900 ± 10.303	12.559 ± 11.257
TDR	0.765 ± 0.283	0.592 ± 0.312	0.679 ± 0.296	0.692 ± 0.292

*Data for pathologic stage were unavailable for 2 patients.

†Data for histologic invasiveness were unavailable for 1 patient.

**P value < 0.05 compared with EGFR-negative patients.

#### Comparison between groups with distinct molecular biomarkers

No significant demographic differences were found between the two molecular biomarker groups. Interestingly, nGGOs with *ALK* rearrangement were associated with significantly higher pathologic stage and larger maximal and solid diameter in comparison to nGGO lesions with *EGFR* mutation, but not in TDR. All *ALK*-positive nodules were classified as IA, but this trend was not significant due to the relatively small sample size (Table 5).

**Table 5:** Clinicopathological characteristics according to molecular biomarkers in nGGO

	***EGFR***	***ALK***	P value*
N	119	6	
Age	63.50 ± 9.11	60.00 ± 12.05	0.571
Sex	M:F = 43:76	M:F = 2:4	0.889
PYR	5.805 ± 14.426	0.750 ± 1.173	0.942
Pathologic stage	117†	6	0.001
0	6	0	
IA	87	1	
IB	18	3	
IIA	1	1	
IIB	2	0	
IIIA	3	1	
IIIB	0	0	
Nodal involvement	5	2	0.003
Histologic invasiveness	118**	6	0.351
AIS	6	0	
MIA	11	0	
IA	101	6	
Maximal diameter	22.387 ± 9.876	33.583 ± 13.736	0.032
Solid diameter	11.133 ± 11.229	23.217 ± 16.906	0.032
TDR	0.702 ± 0.295	0.533 ± 0.327	0.225

*P value: EGFR vs. ALK.

†Data for pathologic stage were unavailable for 2 patients.

**Data for histologic invasiveness were unavailable for 1 patient.

#### Comparison of EGFR mutation and ALK rearrangement rate in GGO nodules to previous studies of a large cohort of adenocarcinomas

The prevalence of *EGFR* and *ALK* mutations in GGO nodules in this study was compared to previous reports of adenocarcinoma of all types. As summarized in Table 6 the *ALK* rearrangement rate (2.8%) in this study was quite low. We previously reported an *ALK* rearrangement rate of 6.8% in all types of adenocarcinoma [23]. Other reports from Korean institutes showed higher rates of *ALK* rearrangement [5.4% [25] and 20.4% [26]]; however, no significant difference was found in *EGFR* mutation rate.

**Table 6:** Prevalence of biomarker mutations in previous large population studies of lung adenocarcinoma

**Molecular biomarker**	**Author (reference)**	**Frequency**	**Total number**	**Population**	**Remarks**
***ALK***	Paik et al. [23]	6.8%	395	Korean	Surgically resected
	Choi et al. [25]	5.4%	331	Korean	Underwent FDG-PET
	Koh et al. [26]	20.4%	221	Korean	Advanced disease
	Takeuchi et al. [31]	3.9%	1121	Japanese	Surgically resected
	Fukui et al. [29]	3.9%	720	Japanese	Surgically resected
	Wang et al. [32]	8.6%	151	Chinese	Advanced disease
	Rodig et al. [30]	5.6%	358	American	Surgically resected, partially
	This study	2.8%	217	Korean	nGGO only
***EGFR***	Choi et al. [25]	47.1%	331	Korean	Underwent FDG-PET
	Kim et al. [46]	43.5%	200	Korean	-
	Sun et al. [49]	53.1%	358	Korean	-
	Kosaka et al. [47]	49.1%	224	Japanese	Surgically resected
	Uramoto et al. [50]	37.8%	437	Japanese	Surgically resected
	Huang et al. [45]	38.1%	858	Chinese	-
	Liam et al. [48]	39.5%	812	Malaysian	-
	This study	54.8%	217	Korean	nGGO only

## Discussion

Lung cancer, in its early stage, can present as nGGOs on chest CT. Lung adenocarcinoma with growth patterns involving the alveolar septum and a relative lack of acinar filling shows GGOs on chest CT, and a high GGO proportion is correlated with good prognosis [27]. Pathology of GGO nodules has shown that the proportion of GGO in nodular adenocarcinomas decreases through the AAH-AIS-MIA-IA pattern of progression [28], and that GGO nodules must undergo *in situ* changes, since AIS (formerly called BAC) and precancerous lesions such as AAH correspond to pure GGO [15].

The clinicopathologic, radiologic, and molecular biological characteristics of nGGOs are important for our understanding of the mechanism of carcinogenesis and for predicting the chemotherapeutic response. Since the introduction of molecular targeting agents, many groups have studied the *EGFR* mutation status of nGGOs, but there is little data on *ALK* rearrangements in nGGOs. *EGFR* mutations are frequently found in the early stages of nGGO, such as in AAH and AIS, and play an important role in the pathogenesis of adenocarcinoma with GGO patterns. However, the role of *ALK* rearrangement, another potent driver mutation in adenocarcinoma, has not been described in GGO nodules.

In this study, we investigated the frequencies and clinicopathological characteristics of driver mutations, focusing on *ALK* rearrangement in resected adenocarcinoma with GGO patterns. To our knowledge, this is the largest comprehensive analysis of lung cancer presenting as GGO nodules. We included lung cancer nodules exhibiting any amount of GGO regardless of its size, thereby investigating the molecular biomarker status of lung cancer at early stages.

Adenocarcinoma with *ALK* rearrangement is usually found in younger, female patients who have light to no smoking history, and has been reported to have acinar, papillary, cribriform, and signet-ring patterns. The radiological characteristics of lung cancer with *ALK* rearrangement have hardly been studied, and there is a lack of data concerning the role of *ALK* rearrangement in nGGO lesions. In one study, Fukui et al. reported that no GGO nodules were found in patients with *ALK* rearrangement while 50% of adenocarcinomas that did not have *ALK* rearrangement also had GGO nodules and also *EML4-ALK*-positive tumors mainly exhibited a solid pattern on CT [29].

In this study, the proportion of *ALK*-positive nGGO lesions was significantly lower (2.8%) than that obtained in previous studies of a large cohort of adenocarcinomas (3.9-20.4%) (Table 6) [23,25,26,29-32], and was significantly lower than the 6.8% of 395 resected adenocarcinoma patients in our previous study, which included all types of curatively resected adenocarcinoma [23]. This could be indirect evidence of the lower incidence of *ALK* rearrangements in adenocarcinomas with GGO patterns compared to adenocarcinomas of all types.

It is well known that *ALK*-positive adenocarcinoma is likely to present a signet-ring cell or cribriform pattern and abundant mucin production on histological analysis [33,34]: *ALK*-positive lesions are observed as a solid, rather than a GGO, nodule [29,35,36]. This explains the low proportion of *ALK*-positive patients in this study, which focuses on nGGOs. Fukui et al. studied the radiologic characteristics of 28 *ALK*-positive adenocarcinomas and revealed no GGO portion [29] and another report on CT characteristics of ALK rearranged advanced NSCLC from Japan also report low frequency of ALK rearrangement (one among 36 cases) [36], consistent with our findings.

We revealed that maximal diameters and the solid portion of nGGOs with *ALK* rearrangement were significantly larger than were those without *ALK* rearrangement. All nGGOs with *ALK* rearrangement were IA (invasive adenocarcinoma) with acinar predominant subtypes (n = 3) and three with cribriform pattern. Patients with *ALK*-positive lesions showed more advanced pathologic stages than those with *EGFR*-positive GGOs. Therefore, we suggest *ALK* rearrangement is associated with cellular and histological type as well as clinical aggressiveness.

Several studies have revealed that adenocarcinomas with *ALK* rearrangement have more lymph node metastases [23,25]. Combined with the radiological characteristics discussed above, the *ALK*-positive adenocarcinoma seems not to follow the stepwise carcinogenesis pattern of AAH-AIS-MIA-IA, but to grow rapidly and bypass the phase of lepidic growth. This assumption is consistent with the histological analysis of *ALK*-positive adenocarcinomas showing lower frequencies of lepidic growth and AAH/BAC (AIS) in the background of *ALK*-positive lung adenocarcinomas [35].

Distinct subsets of adenocarcinoma with morphologic differentiation to type II pneumocytes, Clara cells, or non-ciliated bronchioles are thought to originate from the terminal respiratory unit (TRU), and *EGFR* mutation is involved with early-stage carcinogenesis of TRU-type adenocarcinoma [5,37]; nGGOs appear to be another marker of TRU-type adenocarcinoma [5].

Thyroid transcription factor-1 (TTF-1) is a marker of TRU-type adenocarcinoma [37,38], and two studies concerning 11 and 12 *ALK*-positive patients each revealed TTF-1 positivity in all *ALK*-positive adenocarcinomas [26,39]. This finding suggests that this subtype of adenocarcinoma may have TRU-origin histogenesis [39]. However, the low proportion of GGO with *ALK* rearrangement and the advanced stage in ALK-positive nGGOs found in this study indicates that it is still possible that this subtype may not follow a process of TRU origin. Further pathologic analysis of morphological characteristics is required.

Because the prevalence of adenocarcinoma with *ALK* rearrangement is low compared to *EGFR* mutation, studies investigating various characteristics of *ALK*-positive lung cancer do not gather enough participants to yield consistent results. Previous studies on a large, unselected population of adenocarcinoma with *ALK* rearrangement reported that patients with *ALK*-positive lung cancer were younger [23,29,30,32], female [23,25,40], and light or non-smokers [23,25,29,30,32,40,41]. We previously reported that *ALK*-rearranged lung adenocarcinomas of all radiologic types showed higher stage at diagnosis and more solid pattern, were more cribriform, and had a closer relationship with adjacent bronchioles [42] and more frequently positive bronchoscopic findings than *EGFR*-positive lung adenocarcinoma [43], which suggested more proximal origin of ALK rearranged lung adenocarcinoma than EGFR-positive adenocarcinoma. These findings were consistent with low frequency of ALK rearrangement in nGGOs which presented in peripheral location.

We found no correlation between age, sex, smoking status, and *ALK* positivity, probably due to the small number of *ALK*-positive patients and the weak representation of adenocarcinoma, since we enrolled only patients with nGGOs.

We found that *EGFR* mutation was associated with female, never/light smokers, as expected [44]. The frequency of *EGFR* mutation in nGGOs in this study was 54.8%, which was relatively high in comparison to other, large cohorts of adenocarcinoma [25,45-50] (Table 6). However, we could not predict *EGFR* mutation status by the GGO proportion of nodules or tumor size. *EGFR* mutation status was not associated with pathologic stage, nodal involvement, or histologic invasiveness.

It is interesting that after stratifying *EGFR* mutations in exons 19, 20, and 21, only the mutation in exon 21 (mostly L858R) correlated with female gender and never/light smoking status. This result is consistent with other studies of the characteristics of adenocarcinoma and *EGFR* mutation type [51,52]. The association between *EGFR* and female non- or light smoker may be limited to *EGFR* mutation in exon 21.

According to large cohort studies, EGFR mutations and *ALK* rearrangements are mutually exclusive. However, several cases of co-incident *EGFR* mutation and *ALK* rearrangement have been reported, most of which demonstrated good response to EGFR tyrosine kinase inhibitors [32]. In our study, which recruited participants at the early stage of adenocarcinoma, these molecular biomarkers were mutually exclusive. It is thought that they act through different mechanisms in early carcinogenesis.

The major strength of study is that it is the largest cohort concerning lung cancer with nGGOs. All nodules were resected by curative surgery, which reinforced the accuracy of pathologic and molecular diagnoses of the surgical specimens. Although we collected enough GGO nodules with *EGFR* mutations in exons 19 and 21, we could not collect sufficient numbers of samples with *ALK* rearrangement due to the inherent limitation that adenocarcinoma with *ALK* rearrangement tends to present as solid nodules in chest CT.

## Conclusions

*ALK* rearrangement is rare in lung adenocarcinoma presenting as nGGOs and is associated with a more advanced stage and larger tumor size, suggesting a distinct origin and an aggressive nature in the progression of lung adenocarcinoma. *ALK* rearrangement may not play an important role in the early pathogenesis of nGGO. It is important to understand the clinicopathological characteristics of nGGOs associated with each driver mutation, as well as their radiologic correlations, when individualizing lung cancer treatments with molecular-targeted therapies.

## Abbreviations

*EGFR*: Epidermal growth factor receptor; *ALK*: Anaplastic lymphoma kinase; nGGO: Nodular ground glass opacity; CT: Computed tomography; SPN: Solitary pulmonary nodule; AAH: Atypical adenomatous hyperplasia; AIS: Adenocarcinoma in situ, MIA, microinvasive adenocarcinoma; IA: Invasive adenocarcinoma; TDR: Tumor shadow disappearance rate; IHC: Immunohistochemistry; FISH: Fluorescent *in situ* hybridization; TRU: Terminal respiratory unit; TTF-1: Thyroid transcription factor-1.

## Competing of interest

The authors state that they have no conflict of interest to disclose.

## Authors’ contributions

SJK and CTL had full access to data, writing, and responsibility for the manuscript. YJL, JSP, YJC, HIY, and JHL assisted with recruitment and critical reading of the manuscript. JHC examined the pathology and analyzed *EGFR* and *ALK* status. HK reviewed the pathologic specimen. TJK and KWL analyzed radiological characteristics of nGGOs. KK and SJ performed surgical resection of nGGOs. All authors read and approved the final manuscript.

## Pre-publication history

The pre-publication history for this paper can be accessed here:

http://www.biomedcentral.com/1471-2407/14/312/prepub
